# Distal Tibiofibular Joint Reconstruction Using Autograft in a Rare Case of Lower Limb Sclerosing/Spindle Cell Rhabdomyosarcoma: A Two-Year Follow-Up

**DOI:** 10.7759/cureus.42869

**Published:** 2023-08-02

**Authors:** Homihidayah Othman, Nor Hazla Mohamed Haflah, Mohamed H Sani, Wan Faisham Wan Ismail, Levin Kesu Belani

**Affiliations:** 1 Orthopaedic and Traumatology, Faculty of Medicine, Universiti Kebangsaan Malaysia, Kuala Lumpur, MYS; 2 Orthopaedics, Faculty of Medicine, Universiti Kebangsaan Malaysia, Kuala Lumpur, MYS; 3 Orthopaedic, Universiti Sains Malaysia (USM), Kota Bharu, MYS; 4 Orthopaedic and Traumatology, Fakulti Perubatan, Universiti Kebangsaan Malaysia, Kuala Lumpur, MYS

**Keywords:** reconstruction of ankle joint, short-term outcome, autografts, spindle cell rhabdomyosarcoma, lower extremity tumour

## Abstract

Sclerosing/spindle cell rhabdomyosarcoma (s-scRMS) is a rare variant of striated muscle tumours. It has been recognised as an individual entity, the fourth subtype of rhabdomyosarcoma in the latest WHO classification. In the paediatric population, it occurs more commonly in the paratesticular area, whereas in adults, it occurs more commonly in the head and neck region. It has distinctive characteristics in terms of its histopathological and immunochemistry findings, which help in accurate diagnosis. The mainstay of treatment is a multimodal approach, i.e., surgery, chemotherapy, and radiation therapy. However, no standard care is still being established internationally for adult cases. In adults, this tumour has a poorer prognosis as compared to children. We describe a patient with s-scRMS of the lower limb who has undergone wide local resection of the tumour with surgical reconstruction of the distal tibiofibular joint with autograft and its two-year outcome.

## Introduction

Rhabdomyosarcoma (RMS) is a type of soft tissue malignancy that is most commonly seen in children and rarely in adults [1]. It is traditionally subdivided into three groups: alveolar, embryonal, and pleomorphic. Two new variants, spindle cell RMS and sclerosing RMS, have been reported in the literature and are recognised by the 2013 WHO classification as the fourth subtype of RMS [2]. In this article, we would like to highlight the distal tibiofibular joint reconstruction using autograft in a case of combined sclerosing/spindle cell RMS (s-scRMS), which has been treated with multimodal therapy consisting of chemotherapy, limb-salvage surgery with distal fibula skeletal reconstruction, and radiation therapy.

## Case presentation

A 44-year-old lady with no significant past medical history presented with a one-year history of right ankle swelling that gradually increased in size. There was a history of trauma, where her feet were hit by a steel bar prior to the onset of the swelling. The patient did not seek medical attention initially as the swelling did not affect her activity of daily living. She subsequently started to feel a dull aching pain four months prior to our consultation and ankle stiffness, which was aggravated by prolonged standing and walking.

One month prior to the first consultation, she began to have pain at night, which was relieved by hanging her leg to the side of the bed. She did not complain of any constitutional symptoms or pain and swelling elsewhere. On examination, the right ankle swelling was firm, with undefined margins located at the posterolateral aspect of the right leg extending down to the lateral ankle. The swelling was not warm nor tender, and the Tinel sign was negative. The distal neurovascular function was intact. However, the ankle range of motion, particularly dorsiflexion, was limited due to pain. There was no inguinal lymph node palpable.

**Figure 1:**
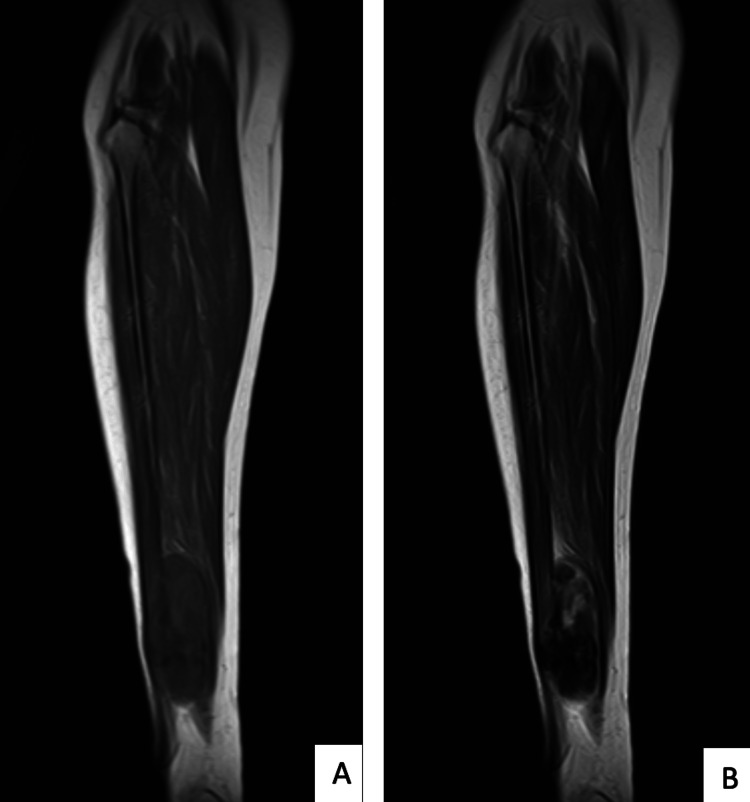
Pre-operative MRI image in coronal cut showing homogeneous isointense in a T1-weighted signal (A) and a heterogeneous hyperintense, well-defined oval-shaped mass on a T2-weighted signal with avid post-contrast enhancement (B) MRI, magnetic resonance imaging

**Figure 2:**
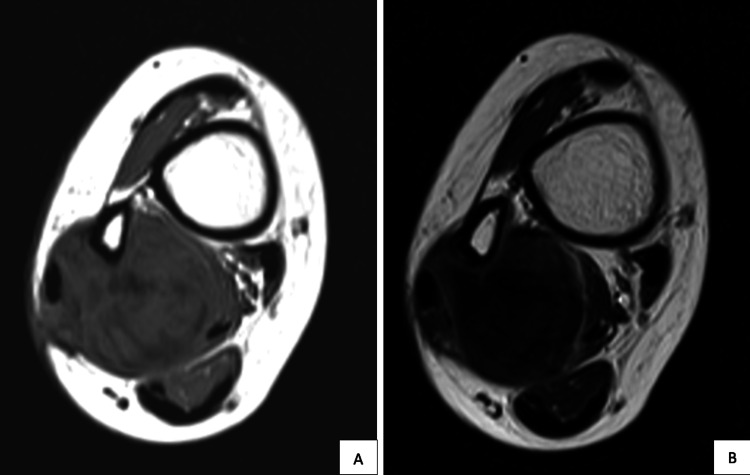
Pre-operative MRI image in axial cut showing the mass located between the flexor hallucis longus, tibialis posterior, and distal soleus muscle in T1- (A) and T2-weighted images (B) MRI, magnetic resonance imaging

The right tibia-fibula radiograph shows no obvious bone lesions. As shown in Figure 1, magnetic resonance imaging (MRI) of the right tibia-fibula shows homogeneous isointense in a T1-weighted signal and a heterogeneous hyperintense, well-defined oval-shaped mass on a T2-weighted signal with avid post-contrast enhancement. The mass was found to arise between flexor hallucis longus, tibialis posterior, and distal soleus muscle, encasing the distal fibula and partially compressing the distal fibular artery and vein, as shown in Figure 2. However, no cortical thinning or periosteal reaction was seen. A core biopsy was performed at the site of the lesion, which showed that the swelling was consistent with s-scRMS. Systemic staging of the patient, which consisted of a computed tomography (CT) scan of the thorax, abdomen, and pelvis, revealed that the swelling was solitary with no distant metastasis.

The patient underwent six cycles of neoadjuvant chemotherapy of the VAC regime, which consisted of IV vincristine, IV actinomycin, IV cyclophosphamide, and IV doxorubicin. A repeat MRI following neoadjuvant chemotherapy of the right tibia-fibula was obtained and showed stable disease in terms of the tumour response. Three weeks after the completion of her sixth cycle of chemotherapy, she underwent a wide local excision of the tumour.

The patient was positioned supine on a radiolucent table under general anaesthesia. A sandbag was placed beneath the ipsilateral buttock to rotate the leg internally. A straight 20-cm longitudinal incision was made in line with the posterior margin of the fibula, starting from proximally. The incision was continued a further 2 cm distally to the tip of the fibula and curved slightly anteriorly. This large incision was done to expose the soft tissue mass that had the encased distal third fibula. A wide local excision was performed, which also included the previous incisional biopsy scar. The encased 15 cm of the distal third fibula was excised using an osteotome and was left with only 3 cm of the tip of the distal fibula. The osteotomy site was decided based on the location of the lesion with a 2-cm margin from the lesion proximally and distally. The 3 cm of the distal fibula was kept, as it served as the attachment of the lateral ankle ligament complex to contribute to the stability of the ankle. 

**Figure 3:**
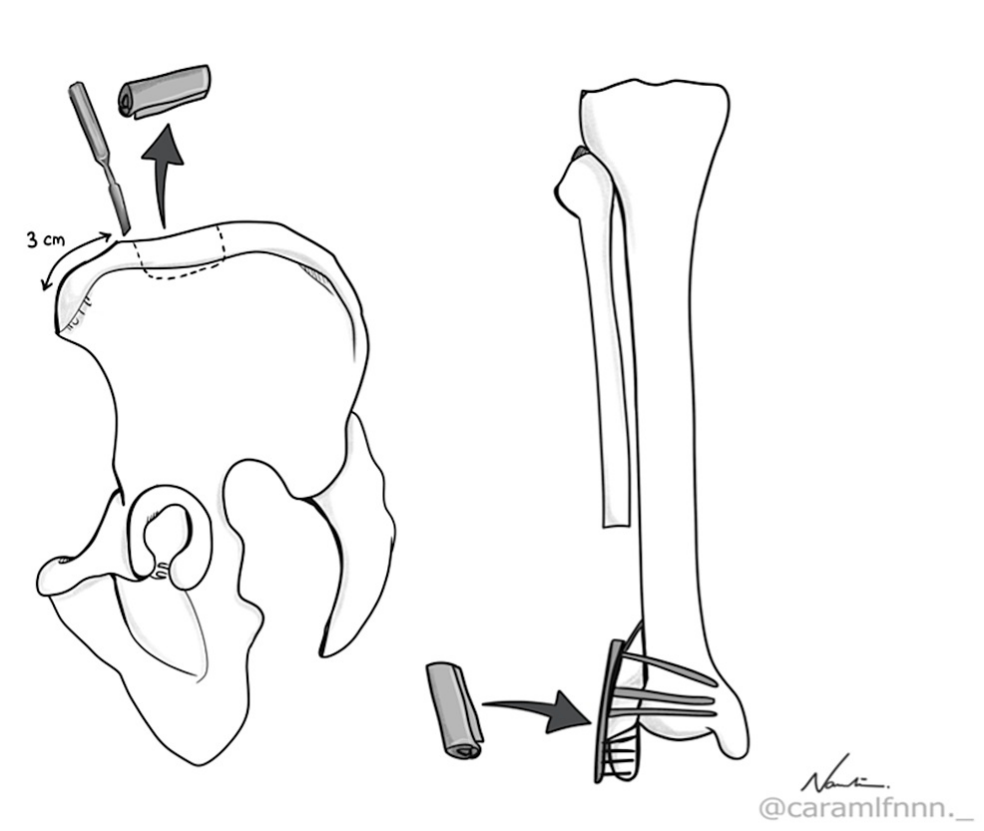
Iliac bone autograft harvesting sketch Tricortical iliac wing autograft is harvested from the inner table of the iliac wing with a 5-cm length and subsequently denuded before reconstructing the distal tibiofibular joint (this figure was sketched personally by one of the authors)

**Figure 4:**
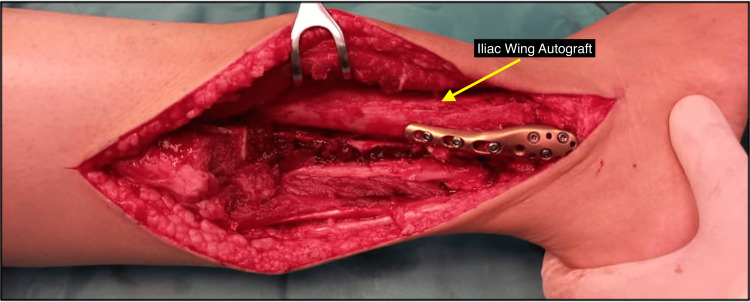
Iliac wing autograft reconstruction of the distal tibiofibular joint The iliac wing autograft is reconstructed at the distal tibiofibular joint with a locking plate

The distal tibiofibular joint was reconstructed in the same surgical setting using a tricortical bone graft that was harvested from the patient’s ipsilateral iliac crest, as represented in Figure 3. The aim of the surgery was to restore the ankle mortise in the hope of creating stability to the distal tibiofibular joint. The bone graft, measured 5 cm, was fixed with a distal fibula locking plate proximally to the lateral cortex of the distal tibia and distally to the remaining tip of the fibula, as shown in Figure 4. Prior to the bone graft fixation, the lateral cortex of the distal tibia was denuded by using bone rongeur and curettage until punctate haemorrhage was seen. This ensures the fusion of the graft with the distal tibia at the syndesmotic region to get a stable ankle joint. The finalised construction was checked under an image intensifier to ensure that the screws did not penetrate the ankle articular surface. After being satisfied with the lateral malleolus restoration, the surgical wound was closed in layers, and an above knee back slab was applied post-surgery. The patient was not allowed to bear any weight for six weeks until she was reassessed during the follow-up.

The patient was discharged one-week post-surgery with no immediate complications. Subsequently, the patient received 25 fractions of radical radiotherapy and, concurrently, three cycles of consolidation chemotherapy with the same VAC regime, which consisted of IV vincristine, IV actinomycin, IV cyclophosphamide, and IV doxorubicin. The histopathological examination of the specimen obtained from the surgery showed cellular fascicles of spindle cells arranged in clusters and cords within a sclerotic collagenous background. The immunohistochemical staining showed that the tumour was positive for desmin and MyoD1 and negative for CD34 and S100.

**Figure 5:**
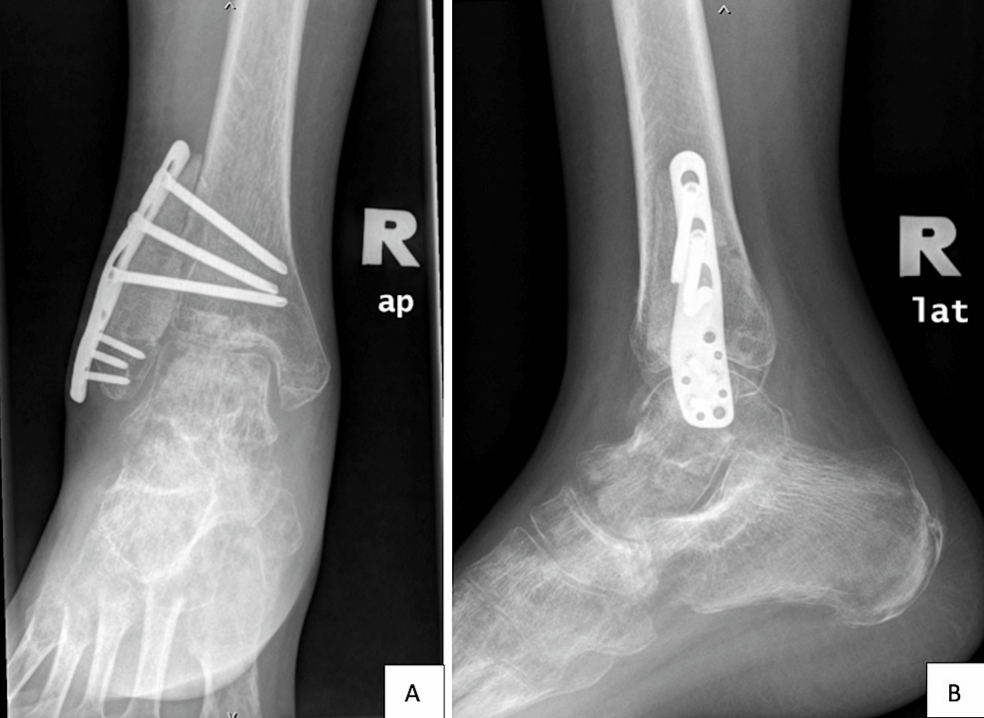
One-year post-operative radiograph showing that the autograft is not united to the tibia bone in the anterior-posterior (A) and lateral views (B)

**Figure 6:**
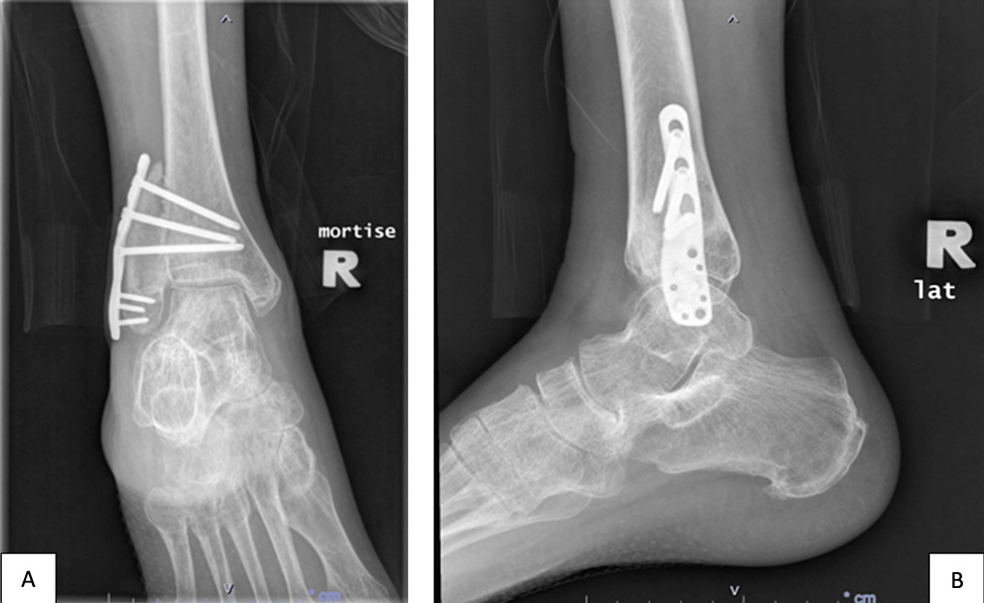
Two-year post-operative radiograph of the right ankle showing the fusion of the graft at the distal tibiofibular joint in mortise (A) and lateral (B) views

One-year post-surgery, the patient was ambulating with minimal pain and stiffness in the ankle joint. The radiograph showed that the autograft was not united to the tibia bone, as shown in Figure 5. At the two-year post-surgery follow-up, she was able to bear weight and ambulate with a walking stick. She was able to dorsiflex (0°-10°) and plantar flex (0°-30°), with minimal inversion but no eversion. There was no recurrence of the swelling. A plain radiograph of the right ankle showed a union of the bone graft with the remaining tip of the distal fibula, as shown in Figure 6. The MRI of the right tibia-fibula and the whole-body PET scan were also performed at one-year post-surgery, which revealed no local recurrence and no distant metastasis.

## Discussion

In adults, RMS is a rare malignancy, and it only makes up <5% of all sarcomas. However, cases of adults affected by the new variant of s-scRMS are more uncommon; hence, only minimal data on clinical course, treatment regime, and prognosis are available in the literature. Similar to other soft tissue malignancies, multimodal therapy is required to manage s-scRMS. Internationally, there is no standard treatment described for adult patients. The chemotherapy medications that are typically administered in this type of sarcoma are vincristine, doxorubicin, cyclophosphamide, actinomycin D, ifosfamide, or etoposide [3]. It is known that RMS is one of the chemosensitive sarcomas. The VAC regime has been established as the standard of care for paediatric RMS. However, it has also been reported that there was more than 50% recurrence or disease progress in s-scRMS, despite the chemotherapy. Moreover, due to its predilection to occur in the head and neck region, it tends to have a poorer outcome. This is because of its proximity to the vital structures and, subsequently, the difficulty of resecting the tumour with a clear margin. 

In our case, the patient had six cycles of neoadjuvant chemotherapy with a VAC regime and showed stable disease after completing her sixth cycle, as evidenced by the MRI findings. Fortunately, our patient’s tumour occurred in her right leg, which allowed a better potential for tumour resection with a clear margin. The tumour encased the distal third of the right fibula. Therefore, it had to be excised with the encased fibula, which left only 3 cm of the distal fibula tip along with the ligaments. The literature proposed that ankle function can generally be preserved when there is a remnant of the distal fibula at least 20 mm above the distal tibiofibular joint. If this is not possible, reconstruction is required because distal fibula resection can cause ankle instability, valgus ankle deformity, and, eventually, secondary osteoarthritis [4-6]. However, distal fibula reconstruction in cases of bone and soft tissue tumours is an uncommon procedure, and currently, only case reports and case series have been described in the literature. According to the literature, several techniques of distal fibula reconstruction have been reported, including vascularised autograft, non-vascularised autograft, allograft, soft tissue reconstruction, and ankle arthrodesis [4-8]. 

Dieckmann et al. reported a case series of wide resection of the entire distal fibula due to malignant bone tumours and employed two techniques of arthrodesis to reconstruct the distal fibula, which were done using screws and retrograde hindfoot nail [4]. In this case series, the Musculoskeletal Tumor Society (MSTS) score of the patients ranged from 80% to 100%, which showed quite a good outcome of the surgery. However, the disadvantage of this technique is the limited range of motion of the ankle. Another technique described by Jamshidi et al. in their case series involved the reconstruction of the distal fibula with an osteoarticular allograft [5]. Their patients required distal fibula resection due to primary bone tumours. However, one 12-year-old patient with osteosarcoma developed a valgus deformity of the ankle due to the ongoing growth of the distal tibia but not in the distal fibula. Therefore, they proposed the use of a longer bone graft for skeletally immature patients to prevent this deformity in the future. Apart from bone reconstruction, the soft tissue reconstruction technique has also been described in the literature. A case series reported by Lamb et al., recently in 2021, employed soft tissue reconstruction of the lateral ankle by using the peroneus brevis tendon [6]. They reconstructed the tendon with a suture anchor in a stellate pattern to the distal tibia, and at follow-ups, both patients had no functional limitations and no major gait disturbance. 

There are also case reports of distal fibula giant cell tumours treated with wide tumour resection, and the skeletal reconstruction technique utilised by the authors was done using a tricortical iliac crest bone autograft [7,8]. In both reports, after 15-year and 18-month follow-up, their patients had a stable ankle joint with a full ankle range of motion. Similar to our patient, the distal fibula reconstruction was done by harvesting a tricortical bone graft from the patient’s iliac crest and fixing it to the remaining lateral malleolus and distal tibia with a locking plate. Post-surgery, the patient underwent 25 fractions of radiotherapy and three more cycles of chemotherapy with the VAC regime. At two-year follow-up, the ankle joint is stable, and the patient can walk with a walking stick. However, due to the adjuvant treatment, she still has a limited range of motion in the ankle. Despite being disease-free for two-year post-surgery, a long-term follow-up with serial imaging is still required because, according to the data available in the literature, the prognosis for s-scRMS is poor, and long-term survival is reduced significantly if the patient develops recurrence [3].

## Conclusions

s-scRMS is a rare subtype of RMS. There is still no standard care being established internationally for adult s-scRMS and the ideal reconstruction technique of the distal tibiofibular joint due to its rarity and limited literature. The advantage of our technique is that it is easily reproducible, and a stable ankle has been achieved. Additional studies may give us more understanding of its clinical course and response and also the outcomes towards certain treatment modalities. 
